# Apicoplast [ā′-pik-ō-plast]

**DOI:** 10.3201/eid3109.241446

**Published:** 2025-09

**Authors:** Hari Shankar, Michal Shahar, Anat Florentin

**Affiliations:** Indian Council of Medical Research, New Delhi, India (H. Shankar); The Kuvin Center for the Study of Infectious and Tropical Diseases and Department of Microbiology and Molecular Genetics, Faculty of Medicine, The Hebrew University of Jerusalem, Jerusalem, Israel (H. Shankar, M. Sahar, A. Florentin)

**Keywords:** Parasites, malaria, Plasmodium falciparum, toxoplasmosis, Toxoplasma, Cryptosporidium, Babesia

The apicoplast is a unique organelle found in obligatory unicellular parasites called Apicomplexa due to a distinguished complex in their apex (top). The phylum Apicomplexa includes human pathogens, such as *Plasmodium* spp. that cause malaria and *Toxoplasma* spp. that cause toxoplasmosis, and prevalent veterinary parasites, such as *Babesia* and *Eimeria* spp.

The apicoplast was first identified in *Toxoplasma* parasites as a relict nonphotosynthetic chloroplast, a plastid, which is a term derived from the Greek *plastos*, meaning molded. The biological, evolutionary, and clinical consequences of that discovery were immediately apparent, and it was given the name apicoplast, a fusion of Apicomplexa and plastid. The name hints at the organelle’s unique evolutionary past. It was formed via secondary endosymbiosis, in which a unicellular protist engulfed another unicellular red alga and its chloroplast. Most Apicomplexan parasites retained that endosymbiont for metabolic purposes but lost all photosynthetic abilities. Few, like the genera of *Cryptosporidium*, lost the entire organelle. Of note, certain nonparasitic organisms related to Apicomplexa, like *Chromera*, still live as marine phototrophs, due to their photosynthetic plastid.

Regardless of photosynthesis, these plastids share similar metabolic pathways, have a small circular remnant genome, and are engulfed by no less than 4 distinct membranes (Figure). Perhaps more than anything, these membranes tell the evolutionary story of the apicoplast; much like a Russian Matryoshka doll, one organism is nested within another.

**Figure:**
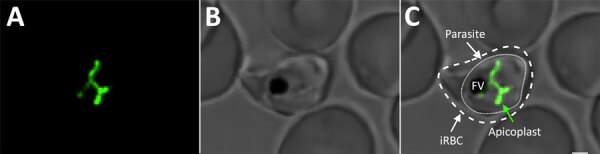
Visualization of the apicoplast organelle inside a malaria parasite. Microscopy image of a *Plasmodium falciparum* transgenic parasite expressing a green fluorescent protein (GFP) fused to a transit peptide, which marks the apicoplast. A) Apicoplast visualized through targeted GFP; B) bright field showing *P. falciparum* parasite in human RBC; C) merged image of GFP and bright field. Outer dashed line depicts the membrane of the iRBC. Inner dashed line depicts the cell membrane of the intraerythrocytic parasite. The intricate green structure is the apicoplast in its elongated phase during the last hours of the intraerythrocytic cell cycle. FV appears as a black sac-like structure. Image taken using a confocal microscope; scale bar indicates 1.25 µm. FV, food vacuole; iRBC, infected red blood cell.
